# Supplementary material for: Agent-based and continuous models of hopper bands for the Australian plague locust: How resource consumption mediates pulse formation and geometry
Source: PLoS Comput Biol. 2020 May 4;16(5):e1007820. doi: 10.1371/journal.pcbi.1007820 (PMC7224576; doi:10.1371/journal.pcbi.1007820)
Supplement: S1 Appendix — Supposing that the stationary-moving transition rates ksm, kms are independent of R, we construct an argument using moments of the resulting density distributions to show that solutions spread indefinitely with a gaussian shape. In particular, there are no coherent pulse solutions with a steep front. (PDF) [file pcbi.1007820.s001.pdf]

Agent-based and continuous models of hopper bands for the Australian plague locust: How resource consumption mediates pulse formation and geometry

Andrew J. Bernoff[1], Michael Culshaw-Maurer[2], Rebecca A. Everett[3], Maryann E. Hohn[4], W. Christopher Strickland[5], Jasper Weinburd[1*]

1 Department of Mathematics, Harvey Mudd College, Claremont, CA, USA
2 Departments of Entomology and Nematology/Evolution and Ecology, University of California Davis, Davis, CA, USA
3 Department of Mathematics and Statistics, Haverford College, Haverford, PA, USA
4 Mathematics Department, Pomona College, Claremont, CA, USA
5 Department of Mathematics and Department of Ecology & Evolutionary Biology, University of Tennessee, Knoxville, TN, USA

All authors contributed equally to this work.
* jweinburd@hmc.edu

Supporting Information

S1 Appendix Resource-Independence: The Telegrapher's Equation
One of our primary assertions is that, in order for the locust population to form a coherent traveling pulse, the two transition rates k_{sm}, k_{ms} must depend on the resource density R. To demonstrate this we will estimate asymptotically the large-time behavior for the population densities in the case with constant transition rates.

Consider the PDE, Eq (9) in the main text, and suppose $k_{sm} \equiv \alpha$ and $k_{ms} \equiv \beta$ (which is equivalent to setting $\eta = \alpha$ and $\theta = \beta$). Then the equations governing the population densities become

$$\begin{aligned} S_t &= -\alpha S + \beta M \\ M_t &= \quad \alpha S - \beta M - v M_x \end{aligned} \qquad x, t \in \mathbb{R} \tag{1}$$

These equations are linear with constant coefficients and can be solved by a variety of means. Physically, they correspond to the probability densities associated to an agent-based model of random switching between stationary and moving states. In this interpretation, α is the probability that an agent transitions from stationary to moving and β is the probability of transition from moving to stationary. As such, we can identify this model as a variant of the *Telegrapher's Equation.* Following previous work (see [1] and references therein), we use the method of moments to determine the large time behavior.

We take initial conditions corresponding to a starting at the origin,

$$S(x,0) = S_0 \delta(x), \qquad M(x,0) = M_0 \delta(x) \tag{2}$$

where S_0 (M_0) is the probability is initially stationary (moving) and δ is the Dirac δ-function. We choose $S_0 + M_0 = 1$, reflecting the fact that this is a probability density.

On average, an agent spends $\frac{\alpha}{\alpha+\beta}$ of its time moving. This leads us to conclude that its average velocity is $c \equiv v\frac{\alpha}{\alpha+\beta}$. This motivates a change of variables $\xi = x - ct$ which

yields

$$\begin{aligned} S_t &= -\alpha S + \beta M + c S_\xi \\ M_t &= \quad \alpha S - \beta M + (c-v) M_\xi \end{aligned} \qquad x, t \in \mathbb{R} \tag{3}$$

In this co-moving frame an agent now moves left with speed c (in the S state) and right with speed $c - v$ (in the M state) but is never stationary.

Solutions to this PDE correspond to probability distributions which can be characterized by their moments. We define the nth moment

$$\mathcal{M}_n(t) \coloneqq \int_{-\infty}^{\infty} \xi^n M(\xi, t)\, d\xi \tag{4}$$

$$\mathcal{S}_n(t) \coloneqq \int_{-\infty}^{\infty} \xi^n S(\xi, t)\, d\xi. \tag{5}$$

Multiplying (3) by ξ^n and integrating yields the equations

$$\begin{aligned} \frac{d\mathcal{S}_n}{dt} &= -\alpha \mathcal{S}_n + \beta \mathcal{M}_n - nc\mathcal{S}_{n-1}(t) \\ \frac{d\mathcal{M}_n}{dt} &= \quad \alpha \mathcal{S}_n - \beta \mathcal{M}_n - n(c-v)\mathcal{M}_{n-1}(t) \end{aligned} \qquad t \in \mathbb{R}^+ \tag{6}$$

A similar calculation yield new initial conditions

$$\mathcal{S}_n(0) = S_0 \delta_{n,0}, \qquad \mathcal{M}_n(0) = M_0 \delta_{n,0} \tag{7}$$

where $\delta_{p,q}$ is the Kronecker δ-function.

When $n = 0$, we obtain the equations governing $\mathcal{S}_0$ and $\mathcal{M}_0$ the probability of a being in state S (or M) at time t,

$$\begin{aligned} \frac{d\mathcal{S}_0}{dt} &= -\alpha \mathcal{S}_0 + \beta \mathcal{M}_0, & \mathcal{S}_0(0) &= S_0, \\ \frac{d\mathcal{M}_0}{dt} &= \quad \alpha \mathcal{S}_0 - \beta \mathcal{M}_0, & \mathcal{M}_0(0) &= M_0. \end{aligned} \tag{8}$$

The solution is

$$\mathcal{S}_0(t) = \frac{\beta}{\alpha+\beta}\left(1 - e^{-(\alpha+\beta)t}\right) + S_0 e^{-(\alpha+\beta)t} \tag{9}$$

$$\mathcal{M}_0(t) = \frac{\alpha}{\alpha+\beta}\left(1 - e^{-(\alpha+\beta)t}\right) + M_0 e^{-(\alpha+\beta)t}. \tag{10}$$

As $t \to \infty$, $\mathcal{S}_0(t) \sim \frac{\beta}{\alpha+\beta}$ and $\mathcal{M}_0(t) \sim \frac{\alpha}{\alpha+\beta}$ as previously advertised.

The first moments $\mathcal{S}_1, \mathcal{M}_1$, when divided by $\mathcal{S}_0$ and $\mathcal{M}_0$, are the centers of mass for the corresponding the probability distributions $S(\xi, t), M(\xi, t)$. To find these, we solve

$$\begin{aligned} \frac{d\mathcal{S}_1}{dt} &= -\alpha \mathcal{S}_1 + \beta \mathcal{M}_1 - c\mathcal{S}_0(t) \\ \frac{d\mathcal{M}_1}{dt} &= \quad \alpha \mathcal{S}_1 - \beta \mathcal{M}_1 - n(c-v)\mathcal{M}_0(t) \end{aligned} \qquad t \in \mathbb{R}^+ \tag{11}$$

and obtain a solution of the form

$$\mathcal{S}_1(t) = (C_1 + C_2 t)e^{-(\alpha+\beta)t} + \frac{\beta v}{(\alpha+\beta)^3}\left[\beta M_0 - \alpha(1 + S_0)\right] \tag{12}$$

$$\mathcal{M}_1(t) = (C_3 + C_4 t)e^{-(\alpha+\beta)t} + \frac{\alpha v}{(\alpha+\beta)^3}\left[-\alpha S_0 + \beta(1 + M_0)\right] \tag{13}$$

where the C_i's are constants depending on $v, \alpha, \beta, S_0, M_0$. We emphasize that each of $\mathcal{S}_1, \mathcal{M}_1$ tend exponentially to a constant as $t \to \infty$. We conclude that, for large times, the centers of mass of the probability distributions are stationary in our co-moving frame. That is, they move with constant speed $c = v\frac{\alpha}{\alpha+\beta}$ as we hypothesized earlier.

We repeat this procedure once more to find the second moments $\mathcal{S}_2, \mathcal{M}_2$, which describe the variance of the probability distributions $S(\xi, t), M(\xi, t)$. The solution is of the form

$$\mathcal{S}_2(t) = (D_1 + D_2 t + D_3 t^2)e^{-(\alpha+\beta)t} + D_4 + \left(\frac{2\alpha\beta^2 v^2}{(\alpha+\beta)^4}\right) t \tag{14}$$

$$\mathcal{M}_2(t) = (D_5 + D_6 t + D_7 t^2)e^{-(\alpha+\beta)t} + D_8 + \left(\frac{2\alpha^2\beta v^2}{(\alpha+\beta)^4}\right) t \tag{15}$$

where the D_i's are again constants. Important here is that the variances of $S(\xi, t)$ and $M(\xi, t)$ are increasing linearly in t. This precludes the possibility of a coherent finite mass traveling pulse for large times.

The Central Limit Theorem can be used to show that the distribution at large times is normally distributed with linearly increasing variances. If we define the variances as

$$(\sigma_S)^2 = \frac{\mathcal{S}_2(t)\cdot\mathcal{S}_0(t) - [\mathcal{S}_1(t)]^2}{[\mathcal{S}_0(t)]^2}, \qquad (\sigma_M)^2 = \frac{\mathcal{M}_2(t)\cdot\mathcal{M}_0(t) - [\mathcal{M}_1(t)]^2}{[\mathcal{M}_0(t)]^2}$$

then, as $t \to \infty$,

$$(\sigma_S)^2 \sim (\sigma_M)^2 \sim \bar{\sigma} t, \qquad \bar{\sigma} = \frac{2\alpha\beta v^2}{(\alpha+\beta)^3}.$$

and

$$S(\xi, t) = \frac{\beta}{2(\alpha+\beta)\sqrt{\pi\bar{\sigma}t}} e^{-\frac{\xi^2}{4\bar{\sigma}t}} + \mathcal{O}(t^{-3/2}) \quad M(\xi, t) = \frac{\alpha}{2(\alpha+\beta)\sqrt{\pi\bar{\sigma}t}} e^{-\frac{\xi^2}{4\bar{\sigma}t}} + \mathcal{O}(t^{-3/2})$$

which reinforces the random walk interpretation of this distribution.

In summary, when k_{sm} and k_{ms} are independent of R, any initial density distribution will spread diffusively. In S2 Appendix, we show that if $\frac{dk_{sm}}{dR} \leq 0$ and $\frac{dk_{ms}}{dR} \geq 0$ and they are not both zero, that there is a traveling pulse solution to the PDE, Eq (9) in the main text.

References

1. Othmer HG, Dunbar SR, Alt W. Models of dispersal in biological systems. Journal of mathematical biology. 1988;26(3):263–298.
