# Supplementary material for: Agent-based and continuous models of hopper bands for the Australian plague locust: How resource consumption mediates pulse formation and geometry
Source: PLoS Comput Biol. 2020 May 4;16(5):e1007820. doi: 10.1371/journal.pcbi.1007820 (PMC7224576; doi:10.1371/journal.pcbi.1007820)
Supplement: S2 Appendix — We prove the existence of traveling wave solutions to the PDE (9) using an invariant region argument. The existence result also provides a selection mechanism; that is, for a given set of parameter inputs there is only one traveling wave. (PDF) [file pcbi.1007820.s002.pdf]

Agent-based and continuous models of hopper bands for the Australian plague locust: How resource consumption mediates pulse formation and geometry

Andrew J. Bernoff[1], Michael Culshaw-Maurer[2], Rebecca A. Everett[3], Maryann E. Hohn[4], W. Christopher Strickland[5], Jasper Weinburd[1*]

1 Department of Mathematics, Harvey Mudd College, Claremont, CA, USA
2 Departments of Entomology and Nematology/Evolution and Ecology, University of California Davis, Davis, CA, USA
3 Department of Mathematics and Statistics, Haverford College, Haverford, PA, USA
4 Mathematics Department, Pomona College, Claremont, CA, USA
5 Department of Mathematics and Department of Ecology & Evolutionary Biology, University of Tennessee, Knoxville, TN, USA

All authors contributed equally to this work.
* jweinburd@hmc.edu

Supporting Information

S2 Appendix Traveling Wave Analysis

Before analyzing the traveling wave solutions to the PDE, Eq (9) in the main text, we simplify notation by rescaling variables into a nondimensional form. We apply the change of variables

$$S = \frac{\alpha}{\lambda}\tilde{S}, \qquad M = \frac{\alpha}{\lambda}\tilde{M}, \qquad R = \frac{1}{\gamma}\tilde{R}, \qquad x = \frac{v}{\alpha}\tilde{x}, \qquad t = \frac{1}{\alpha}\tilde{t}. \tag{1}$$

Note that the dimensionless value of the initial boundary condition is $\tilde{R}^+ = \gamma R^+$ and the conserved quantity is now $\tilde{N} = \frac{\lambda}{\alpha}\int_{-\infty}^{\infty} S + M\,dx$. This yields (after dropping the $\sim$'s) the dimensionless equations

$$\begin{aligned} R_t &= -SR \\ S_t &= -k_{sm}S + k_{ms}M \\ M_t &= k_{sm}S - k_{ms}M - M_x \end{aligned} \tag{2}$$

where now

$$k_{sm} = \phi - (\phi - 1)e^{-R} \qquad \text{and} \qquad k_{ms} = \psi - (\psi - \mu)e^{-\omega R} \tag{3}$$

with four dimensionless parameters

$$\phi = \frac{\eta}{\alpha}, \qquad \psi = \frac{\theta}{\alpha}, \qquad \mu = \frac{\beta}{\alpha}, \qquad \text{and} \qquad \omega = \frac{\delta}{\gamma}. \tag{4}$$

Working from this non-dimensional PDE (2), we utilize a standard traveling-wave ansatz. This amounts to assuming that solutions take the form $S(x,t) = S(x - ct)$, and similarly for M, R. Here c is the speed of our moving reference frame for which we use

the spatio-temporal variable $\xi = x - ct$. The value of c is left to be determined in the forthcoming analysis. We obtain the ODEs

$$\begin{aligned} R_\xi &= \frac{1}{c} SR \\ S_\xi &= \frac{1}{c}(k_{sm}S - k_{ms}M) \\ M_\xi &= \frac{1}{1-c}(k_{sm}S - k_{ms}M). \end{aligned}$$

Subtracting the last two equations, we have $cS_\xi - (1-c)M_\xi = 0$. Integrating once, we have the relation

$$cS(\xi) - (1-c)M(\xi) = 0 \tag{5}$$

where we know the constant of integration on the right must be zero by considering the long-time/far-distance limit $\xi \to \infty$. Using Eq (5), we rewrite the ODE above in terms of the total density $\rho(\xi) = S(\xi) + M(\xi)$. We now have an equation amenable to phase-plane analysis

$$\begin{aligned} R_\xi &= \frac{1-c}{c}\rho R \\ \rho_\xi &= \left(\frac{k_{sm}}{c} - \frac{k_{ms}}{1-c}\right)\rho. \end{aligned} \tag{6}$$

In this two-dimensional ODE, we prove the existence of heteroclinic connections that correspond to traveling wave solutions of Eq (2).

Theorem 1 (Existence of Traveling Waves)**.** *For each c such that $0 < \frac{\phi}{\phi+\psi} < c < \frac{1}{1+\mu} < 1$, there exists a one-parameter family of heteroclinic connections in the phase plane. We parameterize the family by R^+. Each connection goes from from $(R^-, 0)$ to $(R^+, 0)$ and corresponds to a traveling wave solution to the PDE (2) that moves with speed c, and has uniquely determined total mass N, and leaves behind remaining resources R^-.*

Proof. Consider the nullclines of system Eq (6). The R-nullclines are given by the lines $R = 0$ and $\rho = 0$. The ρ-nullclines are given by $\rho = 0$ and the vertical line that satisfies

$$K(R) := \frac{k_{sm}(R)}{c} - \frac{k_{ms}(R)}{1-c} = \frac{\phi - (\phi-1)e^{-R}}{c} - \frac{\psi - (\psi-\mu)e^{-\omega R}}{1-c} = 0. \tag{7}$$

The set of all equilibria is exactly the line $\rho = 0$; no other equilibria exist in the interior of the first quadrant $\rho, R > 0$.

Note that

$$K'(R) = \frac{1}{c}\frac{dk_{sm}}{dR} - \frac{1}{1-c}\frac{dk_{ms}}{dR}$$

which is guaranteed to be less than zero for some c as long as $\frac{dk_{sm}}{dR} \leq 0$ and $\frac{dk_{ms}}{dR} \geq 0$ and they are not both zero.

The ρ-nullcline is a vertical line occurring at $R = R^*$ where R^* satisfies $K(R^*) = 0$. We must ensure that there is an interval of R^+ values such that $R^* \in (0, R^+)$. Note that $K(0) > 0$, $K(\infty) < 0$, and $K'(R) < 0$. By the continuity of P as $R \to \infty$, we can apply the Intermediate Value Theorem and guarantee a unique $R^* \in (0, R^+)$ for any large enough choice of R^+.

Fixing R^+ sufficiently large, we proceed with an invariant region argument, see Fig A. Define the rectangle $A = \{0 < R \leq R^*, 0 < \rho < \rho^*\}$ for an arbitrary ρ^* to be

determined below. Note that region A is invariant as ξ decreases. This is simply due to the fact that $R_\xi > 0$ and $\rho_\xi \geq 0$ on all of A. Therefore, any trajectory intersecting the ρ-nullcline $\{R = R^*\}$ must remain in region A as ξ decreases. By the Poincaré-Bendixson Theorem, the trajectory must terminate on some point $(R^-, 0)$ as $\xi \to -\infty$.

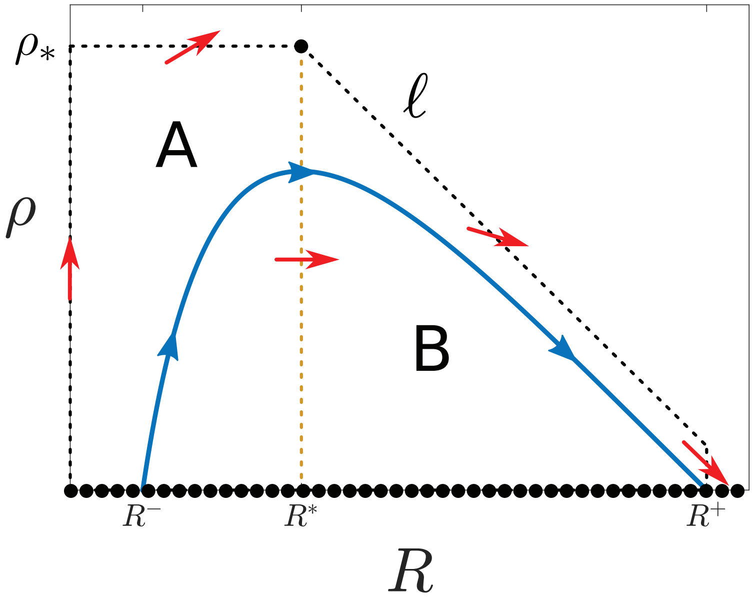

Fig A. The (R, ρ) phase plane with semi-invariant regions A, B bounded by dotted lines including the ρ-nullcline (gold), sample arrows for the vector field (red), and a heteroclinic from $(R^-, 0)$ to $(R^+, 0)$ (blue).

We define region B by choosing the upper boundary as a line segment parallel to and ε-above the stable eigenspace of the linearization of Eq (6) at $(R^+, 0)$ for some small $\varepsilon > 0$. That is,

$$B = \{R^* \leq R < R^+, 0 < \rho < \ell(R)\} \quad \text{where} \quad \ell(R) = \frac{1}{R^+}\frac{c}{1-c}K(R^+)(R - R^+) + \varepsilon.$$

(Note that we may now choose the upper bound of region A to be $\rho^* = \ell(R^*)$.)

All that remains is to show that the stable manifold $\mathcal{W}^s$ of $(R^+, 0)$ is contained within region B until it exits (as ξ decreases) through the vertical ρ-nullcline. Since $\mathcal{W}^s$ is locally tangent to the stable eigenspace near $(R^+, 0)$, we know that it is contained in region B as $\xi \to \infty$. Also $\mathcal{W}^s$ cannot end, as $\xi \to -\infty$, on any of the equilibria composing the lower boundary $\{\rho = 0\}$ of B. This is simply due to the fact that $\rho_\xi < 0$ on the interior of region B so we must have that ρ increases as ξ decreases. Since $R_\xi > 0$ on the vertical line segment between $(R^+, 0)$ and (R^+, ϵ), it is impossible for $\mathcal{W}^s$ to exit B there as ξ decreases. Finally, $\mathcal{W}^s$ cannot exit along ℓ because the slope of the vector field

$$\frac{\rho_\xi}{R_\xi} = \frac{1}{R}\frac{c}{1-c}K(R) \qquad \text{at any point} \qquad (R, \rho)$$

is greater than the slope of ℓ. This follows from the fact that $K'(R) < 0$ and that $R < R^+$ on all of ℓ. In fact, we have shown that, as ξ decreases, no trajectory may exit region B through any part of its boundary other than the vertical ρ-nullcline. □
