# Supplementary material for: Agent-based and continuous models of hopper bands for the Australian plague locust: How resource consumption mediates pulse formation and geometry
Source: PLoS Comput Biol. 2020 May 4;16(5):e1007820. doi: 10.1371/journal.pcbi.1007820 (PMC7224576; doi:10.1371/journal.pcbi.1007820)
Supplement: S3 Appendix — In S2 Appendix we show existence of a traveling wave solution. We now characterize this solution with explicit formulas that relate N, c, R+, and R−. Given any two of these variables and the remaining model parameter inputs, these formulas determine the other two exactly. (PDF) [file pcbi.1007820.s003.pdf]

Agent-based and continuous models of hopper bands for the Australian plague locust: How resource consumption mediates pulse formation and geometry

Andrew J. Bernoff[1], Michael Culshaw-Maurer[2], Rebecca A. Everett[3], Maryann E. Hohn[4], W. Christopher Strickland[5], Jasper Weinburd[1*]

1 Department of Mathematics, Harvey Mudd College, Claremont, CA, USA
2 Departments of Entomology and Nematology/Evolution and Ecology, University of California Davis, Davis, CA, USA
3 Department of Mathematics and Statistics, Haverford College, Haverford, PA, USA
4 Mathematics Department, Pomona College, Claremont, CA, USA
5 Department of Mathematics and Department of Ecology & Evolutionary Biology, University of Tennessee, Knoxville, TN, USA

All authors contributed equally to this work.
* jweinburd@hmc.edu

Supporting Information

S3 Appendix Formulas for N, c, R^+, R^-

In S2 Appendix we show that there exists a traveling wave solution to the PDE model, Eq (9) in the main text. We now characterize this solution with explicit formulas that relate N, c, R^+, and R^-. Given any two of these variables and model parameter values, the following equations determine the other two variables:

$$\frac{I_1}{I_2} = \frac{c}{v-c} \tag{1}$$

$$N\frac{\lambda}{v} = \frac{c}{v-c}\ln\left(R^+/R^-\right) \tag{2}$$

where

$$I_1 = \int_{R^-}^{R^+} \frac{k_{sm}(R)}{R}\,dR, \qquad \text{and} \qquad I_2 = \int_{R^-}^{R^+} \frac{k_{ms}(R)}{R}\,dR. \tag{3}$$

We prove that equivalent equations hold for the nondimensionalized PDE model in S2 Appendix, reproduced here

$$\begin{aligned} R_t &= -SR \\ S_t &= -k_{sm}S + k_{ms}M \\ M_t &= k_{sm}S - k_{ms}M - M_x. \end{aligned} \tag{PDE}$$

Theorem 1. *Given a traveling wave solution to Eq (PDE). Then the quantities N, c, R^+, R^- satisfy*

$$\frac{I_1}{I_2} = \frac{c}{1-c} \tag{4}$$

$$N = \frac{c}{1-c}\ln\left(R^+/R^-\right) \tag{5}$$

where I_1, I_2 are given by Eq (3) with the nondimensional versions of k_{sm}, k_{ms}.

Proof. A traveling wave solution satisfies the ODE in S2 Appendix reproduced below

$$\begin{aligned} R_\xi &= \frac{1-c}{c}\rho R \\ \rho_\xi &= \left(\frac{k_{sm}}{c} - \frac{k_{ms}}{1-c}\right)\rho. \end{aligned} \tag{ODE}$$

Since $R_\xi > 0$ in the first quadrant of the phase plane, we can write ρ as a function of R along any heteroclinic. Thus $\frac{d\rho}{d\xi} = \frac{d\rho}{dR}\frac{dR}{d\xi}$ so integrating along a heteroclinic, we have

$$\int_{R^-}^{R^+} \frac{\rho_\xi}{R_\xi}\,dR = \int_{R^-}^{R^+} \frac{d\rho}{dR}\,dR = \rho(R^+) - \rho(R^-) = 0.$$

We also have

$$\int_{R^-}^{R^+} \frac{\rho_\xi}{R_\xi}\,dR = \int_{R^-}^{R^+} \frac{c}{1-c}\frac{K(R)}{R}dR = \frac{1}{1-c}I_1 - \frac{c}{(1-c)^2}I_2$$

where $K(R)$ is given in Eq (**??**). Therefore we have proved Eq (4).

Dividing the equation for R_ξ in Eq (ODE) by R and integrating the left hand side, we have

$$\int_{-\infty}^{\infty} \frac{R_\xi}{R}\,d\xi = \ln\left(\frac{R^+}{R_-}\right).$$

Meanwhile, the right hand side gives us

$$\int_{-\infty}^{\infty} \frac{1-c}{c}\rho\,d\xi = \frac{1-c}{c}N,$$

proving Eq (5). □

In fact, these equalities can be used to prove monotonicity of the mass-speed relation.

Theorem 2. *Fix R^+. Then the speed c is a strictly increasing function of mass N and a strictly decreasing function of R^-.*

Proof. Let $s = \dfrac{c}{1-c}$. Then Eqs (4)-(5) become

$$s = \frac{I_1}{I_2} \tag{6}$$

$$s = \frac{N}{\ln\left(\frac{R^+}{R^-}\right)}. \tag{7}$$

First, we show $\dfrac{ds}{dR^-} < 0$. Taking the derivative of Eq (6), we get

$$\frac{ds}{dR^-} = \frac{I_2\frac{dI_1}{dR^-} - I_1\frac{dI_2}{dR^-}}{I_2^2} = \frac{I_1k_{ms}(R^-) - I_2k_{sm}(R^-)}{R^-I_2^2}. \tag{8}$$

We will show the numerator, call it $\mathcal{I}$, is negative. Dividing $\mathcal{I}$ by $k_{sm}(R^-)\cdot k_{ms}(R^-)$, we get

$$\frac{I_1}{k_{sm}(R^-)} - \frac{I_2}{k_{ms}(R^-)} = \int_{R^-}^{R^+} \frac{1}{R}\left(\frac{k_{sm}(R)}{k_{sm}(R^-)} - \frac{k_{ms}(R)}{k_{ms}(R^-)}\right)dR. \tag{9}$$

The integrand $\frac{k_{sm}(R)}{k_sm(R^-)} - \frac{k_ms(R)}{k_ms(R^-)}$ is less than zero for $R^- < R < R^+$. To see this, note that at $R = R^-$ this integrand is 0. Also we know that k_{sm} is decreasing in R and k_{ms}

is increasing in R, so the first term decreases and the second term (without the negative sign) increases. Then the integrand is indeed negative for all $R > R^-$.

Second, we will show $\frac{ds}{dN} > 0$. Differentiating Eqs (6) and (7) respectively, we get

$$\frac{dS}{dN} = \frac{\ln\left(\frac{R^+}{R^-}\right) + \frac{N}{R^-}\frac{dR^-}{dN}}{\ln\left(\frac{R^+}{R^-}\right)^2} \tag{10}$$

and

$$\frac{dS}{dN} = \frac{dR^-}{dN}\left[\frac{I_1 k_{ms}(R^-) - I_2 k_{sm}(R^-)}{I_2^2 R^-}\right]. \tag{11}$$

Setting these equal to each other, we obtain

$$\frac{dR^-}{dN} = \frac{R^- \ln\left(\frac{R^+}{R^-}\right)}{\ln\left(\frac{R^+}{R^-}\right)^2 \left[\frac{I_1 k_{ms}(R^-) - I_2 k_{sm}(R^-)}{I_2^2}\right] - N}. \tag{12}$$

Substituting Eq (12) into either Eq (10) or Eq (11), we obtain

$$\frac{ds}{dN} = \frac{\ln\left(\frac{R^+}{R^-}\right)\left[I_1 k_{ms}(R^-) - I_2 k_{sm}(R^-)\right]}{\ln\left(\frac{R^+}{R^-}\right)^2 \left[I_1 k_{ms}(R^-) - I_2 k_{sm}(R^-)\right] - N I_2^2} = \frac{\ln\left(\frac{R^+}{R^-}\right)\mathcal{I}}{\ln\left(\frac{R^+}{R^-}\right)^2 \mathcal{I} - N I_2^2}. \tag{13}$$

We have already shown that $\mathcal{I} < 0$. Because $R^+ > R^-$, the numerator is negative. The denominator is also negative, so have shown that $\frac{ds}{dN} > 0$. □
