# Supplementary material for: Agent-based and continuous models of hopper bands for the Australian plague locust: How resource consumption mediates pulse formation and geometry
Source: PLoS Comput Biol. 2020 May 4;16(5):e1007820. doi: 10.1371/journal.pcbi.1007820 (PMC7224576; doi:10.1371/journal.pcbi.1007820)
Supplement: Attachment — Submitted filename: ResponseRev2.pdf [file pcbi.1007820.s005.pdf]

Dear Reviewer #2,

Thank you for your careful reading of our manuscript and for your particular scrutiny of the biological underpinnings and assumptions. We admit that our team has a strong leaning towards computation and mathematics; we believe the paper now is better connected to the biological literature and field/lab observations. We were absolutely fascinated by the recent study Dkhili et al (2019) which you brought to our attention (it appeared shortly before our submission). We have refined and clarified our biological assumptions, our modeling hypotheses and the goals of our paper.

We have copied your enumerated concerns below and provided direct responses in blue. Revisions spawned from your concerns essentially amount to a rewriting of the following sections of the paper:

- Introduction
- 1.1 Basic Assumptions
- Discussion

Our models, analysis, and results are largely unchanged, although the interpretation has been refined.

We believe that this additional attention has greatly improved the paper, for which we wish to thank you again.

Best,
Jasper Weinburd
(on behalf of the authors)

Reviewer #2 concerns:

1. Does local food abundance indeed regulate the propensity of the locust to move or stand? This idea has been recently tested experimentally by Dkhili et al, “Effects of starvation and vegetation distribution on locust collective motion”. Journal of Insect Behavior, 1-11 (2019). In particular, the authors find that, while starved animals stop and eat before initiating marching activity, ”The results did not show any effects of vegetation distribution on the oriented motion and mean speed”. In other words, feeding and marching are not coupled – starved animals ate first and only then marched, at least in the desert locust studied by Dkhili et al. In contrast, line 33 of the current manuscript states that “While marching, they consume large quantities of green biomass” and cite [11]. Looking through [11], I did not find any report indicating that the Australian plague locusts feed during marching. However, I would be happy to be corrected. Therefore, the authors should, in the very least, make it clear that the main assumption underlying the model, that the transition probabilities p_{sm} and p_{ms} depend on R, are hypotheses that have not been verified experimentally for the Australian plague locusts or are (at best) species specific (and provide a reference).

Thank you for bringing the recent paper of Dkhili et al (2019) to our attention. We have incorporated the experimental results into our modeling assumptions. In particular, the authors find that hoppers in both the starved and fed groups “stopped their oriented movement and moved towards the vegetation to eat once it was introduced”. This supports our assumption that the propensity of the locusts to stop marching depends on local food abundance (see line 156). Furthermore, the authors find that fed hoppers ate only about 50% of the introduced vegetation and “... resumed their movement after [a] short resting period despite the presence of vegetation.” This suggests that the relationship between food and collective, oriented motion is not a simple one: locusts may display collective marching

in the presence of some food. An added complication is that data suggests locust hunger is integral to this relationship. However in our model locusts move through a field of rich food resources and we surmise that locust hunger does not vary as drastically as in experiments with starvation, see lines 180-184.

With regard to our claim that “While marching, they consume…”, we apologize for our imprecision and for the inaccurate citation -- thank you for catching both. We do not claim that individuals eat while crawling; instead, we meant that during long periods (hours) of collective marching, at any given time some fraction of individuals in the hopper band may cease moving and feed (then rest). We have clarified the language and included correct citations in lines 42-46. Additionally, we have pointed the reader to photographic evidence in a number of sources, line 23.

In summary, our model of the transition probabilities p_{sm} and p_{ms} provides a possible explanation of the relationship between collective motion and food. Our explanation is based on field observations [4,10] , lab experiments [19], and foraging theories [37, 38], see lines 154-174. The work of the paper is in determining whether this explanation can reproduce behaviors observed in the field.

2. Do standing animals always eat? To the best of my knowledge this is not true, neither in the lab, nor in field studies. See again, Dkhili et al who report a post-prandial resting period for desert locusts. Thus, the stop-and-go movement pattern may only be interpreted as coarse-grained behavior of moving and standing+eating periods. In particular, the parameter fit from arena experiments without food may not be appropriate here.

We agree and apologize for not robustly addressing this complexity in our first version. We now mention the post-prandial period throughout the paper on lines 47, 136-140, 170-174, 238, and 408. Discussing the limitations of this approach (starting line 171) we write: “After feeding locusts exhibit a post-prandial period of inactivity which extends in proportion with amount consumed [19-21]. Our assumption about this transition rate [from stationary to moving] reflects a longer period of inactivity when resources are plentiful and larger amounts are therefore consumed.” capturing the coarse-grained nature of our assumptions.

When food levels are low, resources R are barely consumed (because of the assumptions explained in #3, below). In this situation, our model still allows for a significant portion of the swarm to be in the stationary state. That is, rich food resources are not a necessary ingredient for individual locusts to stop movement. This is reflected in our choice to match the $R \to 0$ limits of the transition rates to arena experiments with no food.

3. Why is the food consumption rate proportional to R? The authors hypothesize that when food concentration is low, the locust need to spend time searching for it (in the direction perpendicular to motion, which is not modelled explicitly). However, the model assumes a preferred direction, i.e., the locust “know” that the food is at the front. Therefore, why search sideways? Similarly, how do locust “measure” R (to regulate the standing<->moving transition rates)?

Food consumption being proportional to R is consistent with our notion of “foraging” vs. “consuming”. We added detail to explain this on lines 207-214 and also on 403-407:

> “... recall that our ABM places a locust at a grid point which represents a rectangle of physical space… A locust may need to move within this small rectangle to find an individual plant suitable for feeding. Since we track only the resource density in that local rectangle, this search time is simply accounted for in the foraging rate.”

In our model locusts “measure” R by sight and smell nearby vegetation. Rather than “knowing” that the food is at the front, as in chemotactic models [34-36] cited on lines 83-84, locusts in our models have a prefered direction coming from the well-documented social interactions between conspecifics that cause them to align their bodies [2, 4, 8]. We realize that this was not clear before but we hope lines 126-129 rectify our oversight. Again, thank you for helping us to clarify how social alignment is implicitly included in our model.

4. Lack of interaction between the species. It is well documented that the propensity of locusts to move and align their direction with others is highly dependent on their conspecifics. While the authors allude to that in the discussion, it is not clear why this basic property, which has been suggested to be pivotal to locust collective motion [35, 1 and many more] is ignored.

We have partly addressed this in the end of our answer to #3, above. Hopefully we have now made clear that we do include alignment to conspecifics, albeit implicitly, on lines 126-129. We have not included additional additional “social” interactions (such as attraction, repulsion or collision avoidance) because they are outside the scope of the minimal models of this paper, see lines 110-113:

> “Our models are minimal in the sense that we include only the effects necessary to investigate the main question: *Can resource-dependent locust behavior drive the formation of a dense front and the propagation of hopper bands?*”

In other words, prompted by the observations and aerial photographs of [10-13] cited on lines 22-23, our modeling assumption is that the effects of these other social interactions are not the *primary* driver of dense fronts in locust hopper bands. Simultaneously, “We acknowledge that the efficacy of this model may be improved by adding social interactions” on line 187.

Minor comments:

1. Problem with the sentence on line 41.

Our apologies, this should have been caught when proof reading. We fixed this when we revised the Introduction.

2. Line 249: The scheme has to compute derivatives upstream.

Yes, indeed. We now explicitly state this and added an explanation as to why our method is equivalent. See equation above line 304.

3. Fig 1: Is this taken from experiment?

We added in the text that this is a “schematic”, line 22. Also we added specific citations to aerial photos in Hunter et al [10, Fig 2] and others, line 23.

4. Fig 3: It is instructive to note that the mean speed is simply vM/N.

It is true that the instantaneous mean speed of the collective swarm at time t_m is v*sum_{x_n} M_{n,m}/N. However, the dotted line in the figure c=5.3 x 10^{-3} m/s is instead the average of these instantaneous mean speeds over all times t_m after transients subside (i.e. for sufficiently large t_m). Since this is a bit subtle, we chose not to include the explanation in the figure caption.

5. I suggest discussing the effect of noise/diffusion in the discussion section.

We have added some comments regarding noise and diffusion in the discussion, mainly as a possible extension of the model. Lines 799-803.
